# Increasing O-GlcNAcylation Level on Organ Culture of Soleus Modulates the Calcium Activation Parameters of Muscle Fibers

**DOI:** 10.1371/journal.pone.0048218

**Published:** 2012-10-24

**Authors:** Caroline Cieniewski-Bernard, Valerie Montel, Serge Berthoin, Bruno Bastide

**Affiliations:** 1 Université Lille Nord de France, Université de Lille 1, Laboratoire Activité Physique, Muscle et Santé, EA4488, IFR114, IRP2B, Villeneuve d’Ascq, France; 2 Université Lille Nord de France, Université de Lille, Villeneuve d’Ascq, France, 2, Laboratoire Activité Physique, Muscle et Santé, EA4488, IFR114, IRP2B, Villeneuve d’Ascq, France; Duke University Medical Center, United States of America

## Abstract

O-N-acetylglucosaminylation is a reversible post-translational modification which presents a dynamic and highly regulated interplay with phosphorylation. New insights suggest that O-GlcNAcylation might be involved in striated muscle physiology, in particular in contractile properties such as the calcium activation parameters. By the inhibition of O-GlcNAcase, we investigated the effect of the increase of soleus O-GlcNAcylation level on the contractile properties by establishing T/pCa relationships. We increased the O-GlcNAcylation level on soleus biopsies performing an organ culture of soleus treated or not with PUGNAc or Thiamet-G, two O-GlcNAcase inhibitors. The enhancement of O-GlcNAcylation pattern was associated with an increase of calcium affinity on slow soleus skinned fibers. Analysis of the glycoproteins pattern showed that this effect is solely due to O-GlcNAcylation of proteins extracted from skinned biopsies. We also characterized the O-GlcNAcylated contractile proteins using a proteomic approach, and identified among others troponin T and I as being O-GlcNAc modified. We quantified the variation of O-GlcNAc level on all these identified proteins, and showed that several regulatory contractile proteins, predominantly fast isoforms, presented a drastic increase in their O-GlcNAc level. Since the only slow isoform of contractile protein presenting an increase of O-GlcNAc level was MLC2, the effect of enhanced O-GlcNAcylation pattern on calcium activation parameters could involve the O-GlcNAcylation of sMLC2, without excluding that an unidentified O-GlcNAc proteins, such as TnC, could be potentially involved in this mechanism. All these data strongly linked O-GlcNAcylation to the modulation of contractile activity of skeletal muscle.

## Introduction

Many reports highlight the important roles of O-linked-N-acetylglucosaminylation (O-GlcNAcylation, a nuclear and cytosolic modification of proteins by a single monosaccharide, the N-acetyl-D-glucosamine) in nearly all the cellular processes. In this way, since its discovery in 1984 [1], O-GlcNAcylation was shown to be alternately implicated in transcription, in nuclear transport, in mRNA stability, in the regulation of proteasome, in nutrient sensoring, or in the modulation of signalling pathways [2], [3], [4]. There is also an increasing interest in O-GlcNAcylation since some data strongly associate the O-GlcNAcylation dysregulation and the etiology of various pathological disorders such as Alzheimer’s disease, type-2 diabetes, cancer or cardiovascular disorders [5], [6], [7]. Recent data suggests that O-GlcNAc appears to be a regulator of the cellular stress response [8], acute increases being protective in models of acute vascular injury, trauma haemorrhage and ischemia reperfusion injury [9], [10], [11], [12], [13]. In contrast to these studies, O-GlcNAc has also been implicated in the development of hypertension and type II diabetes, leading to vascular and cardiac dysfunction [14], [15] suggesting that chronic elevation of O-GlcNAc is deleterious. Thus, chronic disruption of O-GlcNAcase activity in skeletal muscle, which results in a long term increase in O-GlcNAc, has been demonstrated to be associated to the development of muscle atrophy [16]. In the same way, a correlation has been demonstrated between variations in O-GlcNAcylation levels and the development of atrophy after hind limb unloading, suggesting that O-GlcNAc variations could control the muscle protein homeostasis and be implicated in the regulation of muscular atrophy protecting proteins from degradation through the proteasome [17].

Recent reports suggest that O-GlcNAcylation exerts functions as important as phosphorylation in the healthy striated muscle. On the one hand, indeed several reports showed that many key contractile proteins of skeletal and cardiac muscles are O-GlcNAc modified, *i.e.* myosin heavy chains (slow MHCI as well as the fast isoforms MHCIIA and MHCIIB), myosin light chains (essential MLC or MLC1 and regulatory MLC or MLC2), actin, and both isoforms of tropomyosin [18], [19], [20]. By contrast, little is known about the troponin complex, since only cardiac Troponin I (TnI) has been described to be O-GlcNAc modified [20], while there is no data concerning the troponin complex (TnC, TnI and TnT) in skeletal muscle. The sites of modifications have been mapped for some contractile proteins like cardiac and slow myosin heavy chain, actin, cardiac myosin light chains and troponin I [20], [21]. For certain proteins, O-GlcNAcylation occurs on structural regions involved in protein-protein interactions. However, other sites could modify the properties of these proteins and therefore modified the muscle contractile properties; in particular, the O-GlcNAcylated site on actin is close to the domain of interaction with tropomyosin [21]. On the other hand, O-GlcNAcylation, in addition to phosphorylation, may also regulate muscle contractile function [22]. Thus, functional experiments on skinned fibers demonstrated that exposure to free GlcNAc significantly decreased calcium sensitivity (pCa_50_), whereas maximal force (F(max)) and Hill coefficient (n_H_) were not modified in skeletal or in cardiac muscle fibers [19], [20].

In this paper, we further analyzed the role of O-GlcNAcylation in the modulation of the contractile activity of skeletal muscle fibers, after increasing the level of contractile protein glycosylation in muscle fibers by treatment of soleus biopsies with PUGNAc or Thiamet-G, two inhibitors of the O-GlcNAcase. Our data highlight the key role of O-GlcNAcylation as a modulator of skeletal muscle contractile activity, in particular on the calcium activation properties. Proteomic analysis revealed that skeletal muscle TnI and TnT belong to the O-GlcNAc proteome. Moreover, the analysis of the proteins presenting increase in their O-GlcNAcylation after treatment with PUGNAc suggests a key role of the O-GlcNAcylation of MLC2.

## Experimental Procedures

### Biochemicals

Adult male Wistar rats were purchased from Harlan; DMEM culture medium and ProQ Diamond from Invitrogen; insulin from Organon SA; O-(2- acetamido-2-deoxy-D-glucopyranosilidene)amino-N-phenyl-carbamate (PUGNAc) from Carbogen; Thiamet G from Cayman Chemical; all chemicals reagents, cocktail 1 and 2 anti-phosphatases, penicillin, streptomycin, β-N-acetyl-hexosaminidase, anti-IgM-HRP, anti-goat-HRP, anti-actinin, anti-actin, anti-fastTnT, and anti-Tm from Sigma; CTD110.6, anti-desmin and anti-MLC1 from Abcam; RL-2 from Affinity Bioreagents; anti-TnC and anti-MLC2 from SantaCruz; anti-TnI and slow-TnT antibodies were a generous gift from Dr. JP Jin [23], [24]; trypsin from Promega; Zip-Tips C_18_ and PureProteome ProteinG Magnetic from Millipore; α-cyano-4-hydroxy-cinnamic acid from BrukerDaltonics; nitrocellulose sheet from Biorad; ECL and ECL Plus Western blotting detection reagents from Perkin Elmer; hyperfilms Biomax MR and IPG Dry Strip from GE Healthcare; DIG Glycan Differentiation Kit from Roche; peptide:N-glycosidase F from Biolabs.

### Muscle Isolation

The experiments and maintenance conditions of animals received authorization from the Ministry of Agriculture and the Ministry of Education (veterinary service of health and animal protection, authorization 59-00998). Soleus muscles were freshly removed from adult male Wistar rats anaesthetized with an intraperitoneal injection of sodium pentobarbital (60 mg/kg), before animals were euthanized with lethal intraperitoneal injection of pentobarbital.

### Muscle Incubation

The protocol used was quite similar to that described by Arias et al. [25] and modified from Stace et al. [26]. Freshly excised muscles were incubated for 12 hours in dish plates containing 2 ml of DMEM supplemented to bring final concentrations of 5.5 mM glucose, 2.54 mM CaCl_2_, 25 mM NaHCO_3_, 0.6 nM insulin, 0.1% BSA, 100 µU/ml penicillin and 100 µg/ml streptomycin, the media being replaced with fresh media every 4 hours. Dish plates were placed at 35°C with 95% O_2_/5% CO_2_ and 95% humidity, with gentle shaking.

To increase the O-GlcNAc level on soleus proteins, 150 µM of O-(2-acetamido-2-deoxy-D-glucopyranosilidene)amino-N-phenyl-carbamate (PUGNAc) or 0.5 µM of 2-(ethylamino)-3aR, 6S, 7R, 7aR-tetrahydro-5R-(hydroxymethyl)-5H-pyrano[3, 2-d]thiazole-6, 7-diol (Thiamet G) were added to supplemented DMEM [25]. Control muscles were incubated in the same conditions except that the media did not contain O-GlcNAcase inhibitors.

### Isometric Tension Determination

T/Pca relation-ships were established as previously described [27].

#### Skinning protocol

Immediately after incubation, muscles were chemically skinned by exposure overnight at 4°C to a skinning solution containing 10 mM MOPS, 170 mM potassium propionate, 2.5 mM magnesium acetate, 5 mM K_2_ EGTA, 2.5 mM ATP [27], added with 50 µM PUGNAc. This procedure permeabilizes the sarcolemmal and transverse tubular membranes and allowed the application of activating solutions of various calcium and strontium concentrations (pCa and pSr, with pCa = −log[Ca^2+^] and pSr = −log[Sr^2+^]) directly to the contractile proteins. The skinned biopsies were stored at –20°C in storage solution (glycerol/skinning solution, 50/50, v/v) added of 50 µM PUGNAc until analysis.

#### Solutions

The composition of all solutions was calculated according to Fabiato computer program [28]. Different solutions were used in the experiments: a washing solution (10 mM MOPS, 185 mM potassium propionate, 2.5 mM magnesium acetate); a relaxing solution identical to the skinning solution; pCa and pSr activating solutions corresponding to washing solution added to various concentrations of free Ca^2+^ or Sr^2+^ from CaCO_3_ or SrCl_2_, respectively, buffered with EGTA and added in proportions to obtain the different pCa values (7.0 to 4.2) or pSr values (5.0 and 3.4). ATP was added to each solution to bring a final concentration of 2.5 mM. At the beginning of the experiment, each fiber was bathed for 20 min in 2% Brij58 in relaxing solution, in order to irreversibly eliminate the ability of the sarcoplasmic reticulum of skinned muscles to sequester and release Ca^2+^.

#### Force measurements and effect of O-GlcNAcase inhibitors on calcium properties

A 5 mm fiber segment isolated from skinned biopsies was mounted in an experimental chamber. On one hand, the fiber was connected to a strain-gauge (force transducer Fort 10, World Precision Instruments). The output of the force transducer was amplified and recorded on a graph recorder (Gould. model 6120) and simultaneously analysed by computer software.

At the beginning of each experiment, the fiber was activated with the pSr 5.0 solution, followed by the application of the pSr 3.4 solution, to verify that tested fiber was slow (16% of soleus constitutive fibers being fast [29]). After washing solution, the fiber was activated at a level P with various pCa solutions (from 7.0 to 4.8, with a step equal to 0.2 pCa units). Each steady state submaximal tension P was followed immediately by a maximum contraction Po ensured by pCa 4.2 solution that contained enough calcium to saturate all troponin C sites. The tensions P were expressed as a percentage of the maximal tension Po, and reported as Tension/pCa (T/pCa) relationships. Finally, the fiber was relaxed in relaxing solution. If force declined during a sustained contraction or decreased by more than 20% during the whole experiment, or if T/pCa was not completely achieved, the fibers were rejected from analysis. Just after force measurements, each fiber was resuspended in 10 µl of Laemmli buffer, and stored at −20°C until analysis.

The following parameters were determined from T/pCa curves: the pCa_50_ value, corresponding to 50% of maximal Ca^2+^ tension responses, which characterize the affinity of the contractile apparatus for Ca^2+^; the threshold for activation by Ca^2+^(pCa threshold) an indicator of the calcium sensitivity of the contractile system; and the steepness of the T/pCa reflecting the cooperativity between the different regulatory proteins within the thin filament. The steepness of the T/pCa was determined by the Hill coefficients n_H,_ calculated according to the following equation: P/Po  =  ([Ca^2+^]/K)^nH^/[1+([Ca^2+^]/K)^ nH^]), where P/Po is the normalized tension and K is the apparent dissociation constant (pK  =  −log K  =  pCa_50_). All parameters were established independently for each fibers. Data were presented as means ± SEM. Differences between means were considered significant when *p*<0.05, according to Student’s *t* test.

### Protein Extraction from Skinned Biopsies

After fiber force measurement, protein extraction from resting biopsies was performed using Dounce-Potter homogenization on ice in cold RipA buffer (10 mM Tris/HCl, 150 mM NaCl, 1 mM EDTA, 1% Triton X-100, 0.5% sodium deoxycholate, 0.1% SDS, pH 7.4), adding with 50 µM PUGNAc, antiproteases and anti-phosphatases. Protein concentration was determined according to Lowry assay.

For some analysis, we performed extraction of the whole proteins or contractile proteins from soleus muscle pulverised in liquid nitrogen. Whole proteins extraction was performed by homogenization of muscle powder in RipA buffer; insoluble material was removed by centrifuging at 13,000 rpm for 10 min at 4°C. In order to specifically study the O-GlcNAc modification of contractile proteins, we performed an enrichment of contractile proteins from a total extract of skeletal muscles. Skeletal muscle powder was homogenized in EDTA buffer (6.35 mM, pH 7.0) and centrifuged at 13,000 rpm for 10 min at 4°C. The pellet was washed twice in a 50 mM KCl buffer and suspended again in RipA buffer.

### Western Blot Analysis

#### Analysis of O-GlcNAc pattern

Proteins extracted from fibers included in force measurement analysis (three fibers) were pooled per lane. Proteins were then transferred on 0.22 µm nitrocellulose sheet. Equivalent total proteins load and quality of transfer were confirmed by Ponceau staining. The detection of O-GlcNAc-modified proteins using the CTD110.6 antibody [30] was performed according to Zachara [31], except that washes were performed in TBS-0.3% Tween-20 instead of 0.05%. Detection was carried out using the ECL Plus Western blotting detection reagents and hyperfilms Biomax MR. After the analysis of the O-GlcNAcylation pattern, nitrocellulose membranes were stripped; the total loss of signal was confirmed by a chemiluminescent revelation. A western blot against actin was finally performed in order to quantify the relative amount of proteins between each lane. A negative control experiment was done by incubating the CTD110.6 in presence of 0.5 M of the N-acetyl-D-glucosamine, the competing sugar.

A total densitometry analysis of the western blots was performed to measure the intensity of O-GlcNAcylation with Quantity One Image analyzer software (Bio-Rad). The O-GlcNAcylation level was normalized to the actin level and to the control signal. Data was presented as means ± SEM. Differences between means were considered significant when *p*<0.05, according to Student’s *t* test.

#### Analysis of glycosylated pattern

For glycosylation pattern analysis, whole proteins and contractile proteins extracted from soleus muscle, as well as proteins extracted from skinned biopsies, were treated or not with peptide:N-glycosidase F (PNGaseF), according to the manufacturer’s specifications. Briefly, proteins were denatured by boiling in denaturing buffer. After the neutralization of SDS by NP-40 and dilution in reaction buffer (50 mM sodium phosphate, pH 7.5), 2500 U of PNGase F were added, and reaction was performed overnight at 37°C.

Proteins were then separated on 12.5% SDS-PAGE and transferred onto nitrocellulose sheet. Glycosylation patterns were analyzed using DIG Glycan Differentiation Kit according to the manufacturer’s specifications. Briefly, specific glycosylation domains were detected after lectin recognition. After binding and washes, detection of digoxigenin-labeled lectins was carried out using anti-digoxigenin antibody, alkaline phosphatase-labeled. Staining reaction occurs on membrane by precipitation of the NBT/BCIP substrate.

### Enrichment of O-GlcNAc Proteins

#### Immunoprecipitation of O-GlcNAc proteins

Immunoprecipitation of O-GlcNAc bearing proteins were performed using the IgG RL-2 antibody [32]. Briefly, 500 µg of proteins extracted from soleus skinned biopsies were diluted in RipA buffer adding with PUGNAc, anti-proteases and anti-phosphatases. Each sample was firstly pre-cleared with protein G coupled on magnetic beads.The non-retained sample was then incubated with RL-2 at a dilution of 1/250, overnight at 4°C under gentle agitation. Protein G coupled on magnetic beads was then added at a final dilution of 1/5 (v/v) for 1 hour at 4°C. Following this incubation, beads were washed four times using PBS-0.1% Tween-20 according to the manufacturer’s specifications. Beads were finally suspended again in Laemmli buffer and boiled at 95°C for 10 min. Magnetic beads were carefully discarded and the remaining soluble fraction, corresponding to O-GlcNAc bearing proteins, was finally analyzed using mono-dimensional gel electrophoresis followed by western blot or mass spectrometry analyzes.

#### Enzymatic deglycosylation of O-GlcNAc proteins

To test the specificity of the immunoprecipitation experiments, negative experiments were performed on samples deglycosylated by 10 UI of β-N-acetyl-hexosaminidase treatment prior to the immunoprecipitation protocol, as described by Zachara [31].

### MALDI-TOF Mass Spectrometry Analysis

#### Monodimensional gel electrophoresis

O-GlcNAc proteins immunoprecipitated using RL-2 antibody were separated on 7.5% or 10–20% gel electrophoresis in order to separate respectively high molecular weight proteins or a larger scale of proteins. Gels were colloidal blue stained, and all well-resolved bands were carefully cut from gels until mass spectrometry analysis.

#### “In gel” trypsin digestion and peptides extraction

Each band excised from gels were submitted to in-gel trypsin digestion as previously described [18] with minor changes. Briefly, gel bands were destained, shrunk with acetonitrile (ACN) and dried down. Proteins were then reduced with DTT and alkylated with iodoacetamide. Gel pieces were washed, shrunk with ACN and dried down. The digestion was performed with trypsin at a concentration of 5 or 12.5 ng/µl as previously described [18].

After digestion, peptides were extracted from gel pieces using successive extraction buffers: 25 mM NH_4_HCO_3,_ 45% ACN/0.1% TFA (twice) and 95% ACN/0.1% TFA. All the extracts were pooled together and dried down in a vacuum centrifuge.

#### Mass spectrometry and databases searching

The dried peptides were suspended again in 0.1% TFA just prior to being desalted using Zip-Tips C_18_. Peptides were directly eluted using a freshly prepared α-cyano-4-hydroxy-cinnamic acid matrix, at a concentration 10 mg/mL in 50% ACN/0.1% TFA and spotted directly onto the MALDI-TOF target. Protein identification was performed using peptide mass fingerprinting on a MALDI-TOF mass spectrometer (Voyager DE-STR PRO) as previously described [18]. Each spectrum was internally calibrated using the monoisotopic mass of the fragments resulting from trypsin autoproteolysis respectively at 842.5100, 1045.5642 and 2211.1046 Da.

Proteins were identified using Protein Prospector (http://prospector.ucsf.edu/), Profound (http://Prowl.rockefeller.edu/) and Mascot (http://www.matrixscience.com/) servers from NCBI and Swiss-Prot databases. The following parameters were used: *rattus norvegicus* species, one missed cleavage site and a mass tolerance setting of 50 ppm. Carbamidomethylation of cysteine and partial chemical modifications such as oxidation of methionine were considered for the queries. The criteria used to accept identifications included the extent of sequence coverage, the number of matched peptides, the percentage of recovered sequence, the Mowse probability score, the mass accuracy and whether the protein appeared as the top candidate. Otherwise, the identification was not considered valid.

### Analysis of O-GlcNAc Level on Specific Contractile Proteins

#### O-GlcNAc level analysis on myosin heavy chain

Twenty µg of proteins extracted from biopsies were separated on 7.5% acrylamide/bisacrylamide (50∶1) SDS-PAGE [33]. After migration, proteins were transferred on nitrocellulose sheet, and the western blot analysis with CTD110.6 antibody was performed according to the protocol described above.

#### O-GlcNAc level analysis on other identified contractile proteins

Five hundred proteins were used for RL-2 immunoprecipitation according to the protocol previously described. The O-GlcNAc bearing proteins were separated on 10–20% gels electrophoresis and transferred onto nitrocellulose membranes. The efficiency of the transfer was confirmed by Ponceau staining. All primary antibodies (directed against TnC, slow TnT, fast TnT, TnI, tropomyosin, actinin, desmin, MLC1, MLC2, actin) were incubated overnight in blocking solution (5% non-fat dry milk in TBS-0.05% Tween-20) at a specific dilution determined according to laboratory preliminary experiments. After washing, secondary HRP-labeled antibodies were incubated for 1 hour at room temperature in blocking solution. Membranes were extensively washed, and detection was carried out using the ECL Plus Western blotting detection reagents and hyperfilms Biomax MR.

The intensity of the bands was quantified with Quantity One Image analyzer software (Bio-Rad). Data was presented as means ± SEM. Differences between means were considered significant when *p*<0.05, according to paired Student’s *t* test. Preliminary experiments were performed in order to determine the linear range of the signal.

## Results

### Effect of PUGNAc-treatment of Soleus on Calcium Activation Properties

#### Calcium activation properties of hyperglycosylated soleus

We performed a short term organ culture of rat soleus muscle in presence of PUGNAc to increase the O-GlcNAc level on proteins. Immediately after incubation, soleus biopsies treated or not with PUGNAc were chemically skinned, leading to the permeabilization of the sarcolemmal and transverse tubular membranes. The skinning protocol enables “a direct access” to motor and regulatory contractile proteins. This procedure allows muscular contraction to be set off by the way of an external application of calcium, in order to determine the calcium activation parameters, and to reveal specific contractile proteins or the O-GlcNAcylation patterns using western blot analysis.

**Figure 1:**
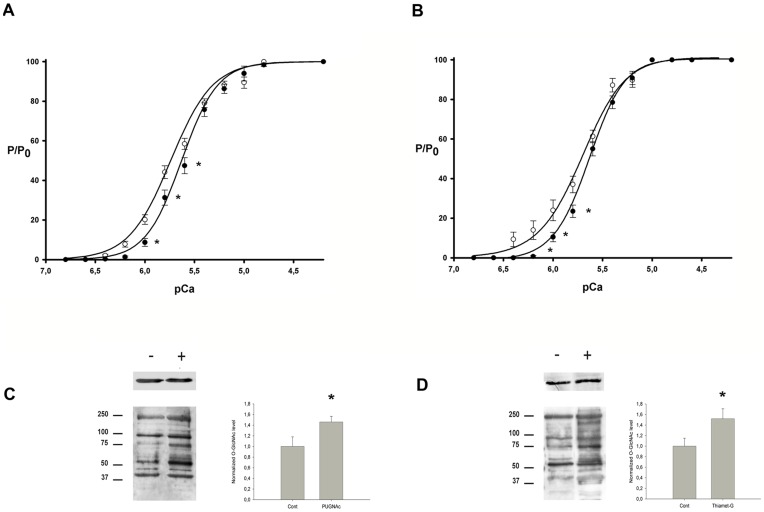
Effect of the increase of O-GlcNAc level on calcium activation parameters of skinned fibers isolated from soleus. (A) T/pCa curves were representative of 13 fibers from untreated skinned biopsies (•) and 26 fibers from PUGNAc-treated skinned biopsies (○). (B) T/pCa curves were representative of 7 fibers from untreated skinned biopsies (•) and 7 fibers from Thiamet G-treated skinned biopsies (○). Data were presented as mean ± SEM. Curves were fitted with the Hill parameters. (C) Proteins from untreated (-) or PUGNAc-treated (+) skinned fibers included in T/pCa relationship analysis in A were separated on 10–20% linear gradient gel electrophoresis and analysed on CTD110.6 western blot (below) or actin (above). Three fibers were pooled per lane. (D) Proteins from untreated (-) or Thiamet G-treated (+) skinned fibers included in T/pCa relationship analysis in B were analysed by western blot using the CTD110.6 antibody (below) or actin (above). Total densitometry analysis of the western blots was performed and normalized to the control signal for PUGNAC- and Thiamet-G-treated fibers respectively; resulting histograms were presented next to the representative blot image. * indicates significant differences between untreated and treated biopsies (p<0.05).

We have determined calcium activation parameters on fibers carefully isolated from control and hyperglycosylated skinned soleus biopsies. Preliminary experiments demonstrated that the calcium activation properties were not modified by the incubation protocol (**figure S1**). We compared the T/pCa relationships from fibers isolated from untreated biopsies (n = 13) and from fibers isolated from PUGNAc-treated soleus biopsies (n = 26); results are presented on Fig.1A, and the corresponding data (*i.e.* pCa_50_, pCa threshold and Hill parameter n_H_) are indicated in table 1. While the maximal tension developed under a saturated Ca^2+^ concentration (pCa_4.2_), 35.05±4.87 mg and 33.01±2.66 mg in untreated and PUGNAc treated biopsies respectively is not modified (data not shown), our data strongly showed that the T/pCa relationships corresponding to fibers isolated from PUGNAc-treated biopsies shifted toward higher pCa value since the pCa_50_ value increased from 5.63±0.03 to 5.74±0.03 for untreated and PUGNAc-treated biopsies respectively (p<0.05). The pCa threshold as well as Hill parameters (n_H_) remained unmodified, as indicated in table 1.

**Table 1:** Activation threshold, pCa_50_ and Hill parameter values of skinned fibers isolated from untreated and PUGNAc-treated soleus.

	Untreated fibers	PUGNAc-treated fibers
**pCa_50_**	5.63±0.03	5.74±0.03*
**Threshold**	6.25±0.06	6.27±0.03
**n_H_**	2.39±0.18	2.05±0.11

* p<0.05 untreated *versus* PUGNAc-treated fibers.

Similar results were obtained when biopsies were incubated in presence of Thiamet G, another inhibitor of O-GlcNAcase (Fig.1B; table 2). Indeed the T/pCa relationships corresponding to fibers isolated from Thiamet-G -treated biopsies shifted toward higher pCa value since the pCa_50_ value increased from 5.64±0.02 to 5.78±0.04 for untreated (n = 7) and Thiamet-G-treated biopsies (n = 7) respectively (p<0.05). The pCa threshold was not significantly modified.

**Table 2:** Activation threshold, pCa_50_ and Hill parameter values of skinned fibers isolated from untreated and Thiamet-G-treated soleus.

	Untreated fibers	Thiamet-G-treated fibers
**pCa_50_**	5.64±0.02	5.78±0.04*
**Threshold**	6.31±0.04	6.45±0.09
**n_H_**	2.51±0.21	2.32±0.23

* p<0.05 untreated *versus* Thiamet-G-treated fibers.

#### Analysis of O-GlcNAcylation pattern on skinned fibers included in T/pCa experiments

We performed a western blot analysis using the CTD110.6 antibody on the fibers isolated from untreated and PUGNAc- or Thiamet-G-treated biopsies and included in T/pCa analysis. To gain a sufficient signal/noise ratio, three fibers were pooled per lane, according to preliminary experiments (data not shown). A representative image of immunoblot was presented on Fig.1C for PUGNAc-treated fibers, and Fig.1 D for Thiamet G-treated fibers. We observed an increase of CTD110.6 immunoreactivity on fibers isolated from PUGNAc- and Thiamet-G-treated soleus, especially for higher molecular weight proteins, while we cannot detect sufficient signals to be analyzed on proteins with a low molecular weight. A total band densitometry analysis revealed a significant increase in the O-GlcNAc signal in PUGNAc- and Thiamet-G-treated fibers by a factor of 1.46 and 1.52, respectively (Fig.1C and Fig.1D).

Taken together, these results indicate that the increase of O-GlcNAcylation on skinned fiber proteins is closely associated with a higher affinity of skinned fibers toward calcium (shift of the T/pCa curve towards the left, as described just above).

#### Analysis of glycosylation pattern of skinned biopsies

**Figure 2:**
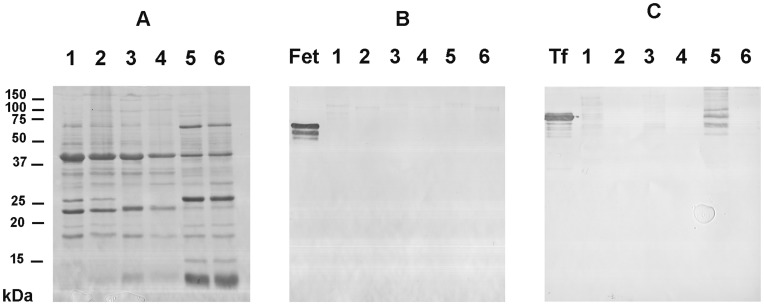
Glycopattern analysis of proteins extracted from skinned biopsies comparing with contractile proteins and whole proteins extracts. Soleus contractile proteins (lanes 1 and 2), proteins extracted from skinned biopsies (lanes 3 and 4) and whole proteins extracted from soleus (lanes 5 and 6) were separated on 12.5% SDS-PAGE and transferred onto nitrocellulose sheet for western blot analysis. Lanes 2, 4 and 6 corresponds to PNGaseF-treated proteins. (A) corresponds to Ponceau staining, (B) to AP-revelation for MAA binding on glycoproteins, (C) to AP-revelation of SNA binding on glycoproteins. Fet (fetuin) and Tf (transferin) correspond to positive glycoprotein markers.

In order to validate that the changes in T/pCa relationship were exclusively due to modulation of O-GlcNAc level, we analyzed the glycosylation profile using lectin recognition of glycosylation domains on proteins treated or not with peptide:N-glycosydase F (PNGase F). Whole proteome extracted from soleus muscle were analyzed using this approach, as well as contractile proteins. Results were compared with proteins extracted from skinned biopsies. Data are presented on Fig.2. The efficiency of the lectin binding of specific moieties was performed by inclusion of positive marker on each gel.

On Fig.2A, Ponceau staining showed protein patterns from contractile proteins extract (lanes 1 and 2), from skinned biopsies (lanes 3 and 4) and whole protein extract (lanes 5 and 6). Peptide:N-glycosidase F treatment (lanes 2, 4 and 6) did not alter the protein patterns. Comparison between whole extract and contractile proteins showed different patterns of proteins; in particular, contractile proteins were enriched in constitutive proteins of thin and thick filaments, *i.e.* motor and regulatory proteins, as well as structural proteins. Protein profiles of proteins extracted from skinned biopsies were quite similar to contractile proteins than whole extracts, but some differences could be also noted between contractile profiles and skinned biopsies; in particular, several proteins are lacking in skinned biopsies compared with contractile protein extracts.

As shown on Fig.2B, no specific binding of Maackia amurensis agglutinin (MAA, indicates sialic acid terminally linked (2–3) to galactose) neither on whole protein extracts nor on contractile proteins. No signal was detected on proteins isolated from skinned biopsies, while positive signal was observed for the glycoprotein fetuin, indicating the efficiency of the lectin binding on glycosylated moieties. The same results were obtained for Galanthus nigra agglutinin (GNA, indicates mannose, terminally linked), Peanut agglutinin (PNA, indicates galactose-β(1–3)-N-acetylgalactosamine) and Datura stramonium agglutinin (DSA, indicates galactose-β(1–4)-N-acetylglucosamine) (data not showed).

The Sambucus nigra agglutinin (SNA, indicates sialic acid terminally linked (2–6) to galactose or N-acetylgalactosamine) showed signals on whole protein extracts as well as on the internal standard corresponding to transferin. These signals disappear on deglycosylated proteins. In contrast, very slight signals were detected on contractile proteins extract, while no signal was detected on proteins extracted from skinned biopsies used for T/pCa relationship establishment.

It is noteworthy that classical N- and O-glycans were not detected on skinned biopsies, sarcolemme being partially eliminated, as well as intracellular membrane, cellular compartments where complex glycans are found are absent on skinned biopsies and so on skinned fibers included in T/pCa relationship analysis. These data argue in favor of an exclusive glycosylation through O-linked-N-acetylglucosaminylation on proteins extracted from skinned biopsies.

### Characterization of O-GlcNAc Bearing Proteins from Skinned Soleus Biopsies

#### O-GlcNAc proteins identification using mass spectrometry analysis

**Figure 3:**
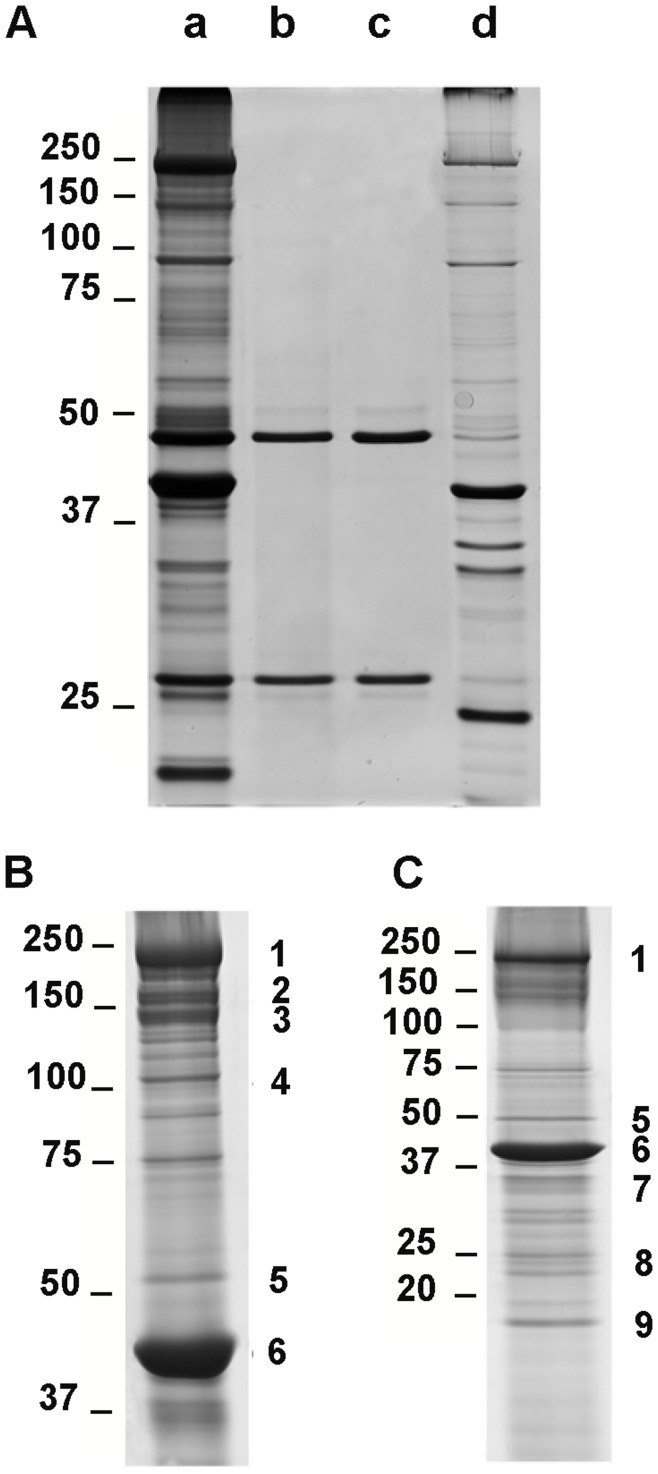
Identification of O-GlcNAc bearing proteins purified from soleus skinned biopsies. (A) corresponds to a silver stained SDS-PAGE of O-GlcNAc proteins purified by RL-2 immunoprecipitation before (a) or after (b) an hexosaminidase treatment; (c) corresponds to immunoprecipitation protocol realized without proteins sample, and (d) to 25 µg of contractile proteins extract. O-GlcNAc proteins were purified by RL-2 immuno-precipitation followed by an electrophoretic separation on 7.5% (B) or 10–20% (C) SDS-PAGE. Gels were colloïdal blue stained. Each well-resolved bands were submitted to a mass spectrometry-based identification. Numbers on the two panels indicates the different identified proteins; note that the corresponding identifications are summarized in table 3.

In order to attempt an identification of O-GlcNAc-bearing proteins, we performed an immunoprecipitation using the RL-2 antibody starting from skinned biopsies; the proteins profiles were presented on figure 3. As shown on Fig.3A, the RL-2 antibody retains a large number of proteins as shown on lane (a) as compared with a contractile proteins extract (lane (d)); the enrichment corresponds exclusively to O-GlcNAc-bearing proteins since we observed the total loss of signals when proteins were deglycosylated by hexosaminidase treatment prior to the immunoprecipitation (lane (b)). The only two observed proteins correspond to the heavy and light chains of immunoglobulins as compared with lane (c), which corresponds to immunoprecipitation protocol without applying a protein extract.

**Table 3:** Detailed list of O-GlcNAc identified proteins from soleus skinned biopsies.

Band number	Accession number	Protein name	Exp. Mw (kDa)	Theo. Mw (kDa)	Number of matched peptides/total peptides	Probability based mowse score; expect	Sequence coverage (%)
**1** **2**	8393807	**Myosin heavy chain 7, cardiac muscle beta** **isoform, slow isoform**	206.42149.96	223.56	56/8870/97	243; 3.3e-20302; 4.2e-26	2731
**3**	149063942	**Isoform CRA_b**	135.53	120.25	34/59	200;6.7e-16	20
**4**	157951643	**Actinin, alpha 2**	97.65	104.34	37/73	232; 4.2e-19	35
**5**	11968118	**Desmin**	49.18	53.42	25/77	210; 6.7e-17	49
**6**	149043182	**Actin, alpha 1, skeketal**	42.28	51.97	14/49	117; 1.3e-7	36
**7**	66730475	**Tropomyosin beta chain**	37.54	32.99	18/90	81; 5.1e-4	42
**8**	6981240	**Myosin light chain, alkali, slow-twitch muscle**	23.34	22.25	11/64	82; 4.0e-4	55
**9**	57580	**Alpha-B crystalline**	19.32	19.94	13/59	130; 6.7e-9	59

Band numbers are assigned to proteins identified from the two gels presented on Fig.3B and 3C; identified proteins are detailed using the NCBI accession number, their generic name and their general function. Exp.Mw corresponds to experimental molecular weight; theo.Mw, to theorical molecular weight. Total peptides correspond to peptides numbers used for research on databases. Probability based mowse scores and expects results were obtained from Mascot database (www.matrixscience.com).

Assignments were made according to UniProt release 2010_07 which consists of: UniProtKB/Swiss-Prot Release 2010_07 of 15-Jun-10 (517 802 entries) and to UniProtKB/TrEMBL Release 2010_07 of 15-Jun-2010 (11 109 684 entries).

The same protocol of immunoprecipitation was applied for enrichment of O-GlcNAc proteins from soleus skinned biopsies, the proteins being separated on 7.5% SDS-PAGE (Fig.3B) or on 10–20% SDS-PAGE (Fig.3C) to have a higher resolution of protein patterns. All well-resolved bands were submitted to a mass-spectrometry-based identification. Next to these gels, we have numbered the identified proteins; table 3 summarizes the identification of the corresponding proteins with their NCBI accession number. In this table were also indicated the function of these proteins, their theoretical and experimental molecular weights, the number of matched peptides per total peptides used for the query on protein identification software, the probability mowse score (from Mascot software) and the protein sequence coverage, which were the principal parameters considered for the accuracy of identification.

Among the O-GlcNAc proteins isolated from soleus skinned biopsies, we have identified contractile proteins previously described to be O-GlcNAc modified, *i.e.* the slow isoform of myosin heavy chain (corresponding to bands 1 and 2 on Fig.3 and in table 3), and actin alpha 1 (band 6), which are the major contractile proteins implicated in muscular contraction, as well as the beta isoform of tropomyosin (band 7), and the essential myosin light chain (band 8), implicated in the regulation of contraction. We have also identified some of the structural proteins of the sarcomere, *i.e*. the small heat shock protein alpha-B crystallin (band 9) and proteins newly identified to be O-GlcNAcylated: actinin alpha 2 (band 4) and desmin (band 5).

We did not identify the regulatory MLC (MLC2) using this approach, while it is known to be O-GlcNAc modified [19], nor the proteins of troponin complex, probably due to their low stoichiometry of O-GlcNAc residues. Thus, we used another strategy presented below to determine first whether troponins were O-GlcNAc modified, and second if the O-GlcNAc level on the identified proteins above was modified in our model of hyperglycosylated soleus.

#### Does troponins (C, T and I troponin) bear an O-GlcNAc moiety?

**Figure 4:**
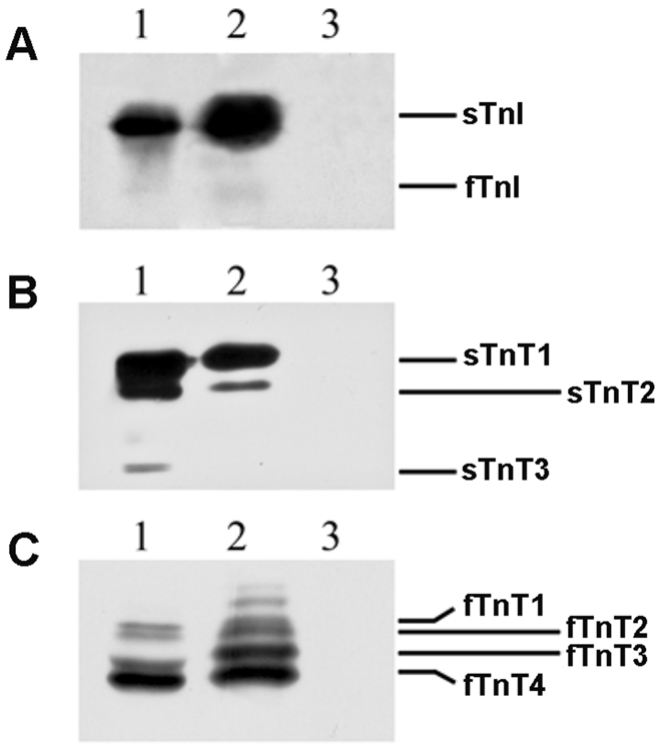
Characterization of O-GlcNAcylation on regulatory proteins of thin filament. O-GlcNAc contractile proteins extracted from soleus were enriched using RL-2 immunoprecipitation protocol. The separation of immunoprecipitated proteins on 10–20% SDS-PAGE was followed by a western blot analysis using antibodies directed against troponin I (A), and slow and fast troponin T (respectively B and C); each isoform was indicated close to the blot. Lane 1 corresponds to whole contractile protein extract from soleus muscle (25 µg of contractile proteins); lane 2 to RL-2 immunoprecipitation protocol; lane 3 to immunoprecipitation experiment performed on hexosaminidase-treated proteins, *i.e*. on deglycosylated contractile proteins. Only the region of the protein of interest is shown.

To determine whether troponins are O-GlcNAc modified in soleus, we performed western blot analysis using antibodies directed against troponins on O-GlcNAc proteins enriched by RL-2 immunoprecipitation. The experiment was realized in parallel on proteins submitted to hexosaminidase treatment prior to immunoprecipitation. Data obtained were presented on figure 4; for each panel, lane 1 corresponds to whole contractile proteins extract from soleus, lane 2 corresponds to O-GlcNAc proteins resulting from RL-2 immuno-precipitation, while lane 3 corresponds to contractile proteins submitted to hexosaminidase treatment prior to immunoprecipitation. As shown on figure 4, some of the proteins of the troponin (Tn) complex are O-GlcNAc modified, *i.e.* TnI (Fig.4A) as well as TnT (Fig.4B and 4C), since we observed signals on RL-2 immunoprecipitated fraction (lane 2) as compared with whole extract (lane 1). No signals were obtained when contractile proteins were deglycosylated prior to the enrichment of O-GlcNAc proteins (Fig.4, lane 3).

The slow isoform of TnI (sTnI), which is the major isoform of TnI in soleus, is O-GlcNAc modified as well as the fast TnI (fTnI) even if its signal remains very low because of the low expression of fast isoform in soleus. Concerning TnT, there are several isoforms presenting a molecular weight from 31 to 36 kDa. In whole protein extract, three slow isoforms (panel B) are resolved and assigned as previously described [34], [35]. In the fraction of proteins retained by RL-2 antibody (lane 2), the sTnT1 was clearly revealed, as well as sTnT2, while no signal was detected for sTnT3. Concerning the fast isoforms of TnT (panel C), several isoforms were separated in whole extract, corresponding to fTnT1, fTnT2, fTnT3 and fTnT4 as previously described [34]. All these isoforms were detected in RL-2 immunoprecipitated proteins, whereas two supplementary isoforms of fTnT appeared on O-GlcNAc proteins fraction, but with a minor expression level comparing with others.

TnC was not identified to be O-GlcNAcylated, using this approach. This suggests that TnC is not an O-GlcNAcylated protein, but of course, we cannot exclude that we were below the detection threshold.

### Quantification of O-GlcNAc Level Variations on Key Contractile Proteins in PUGNAc-treated Soleus

We quantified the variation of O-GlcNAc level on the contractile and structural proteins identified above with mass spectrometry analysis and immunoprecipitation. The quantification was performed using western blot analysis of contractile proteins of interest on O-GlcNAc-enriched proteins from untreated and PUGNAc-treated soleus biopsies (n = 5). This protocol was used to analyze the modulation of O-GlcNAc level on actinin, desmin, actin, tropomyosin, myosin light chains (essential and regulatory) as well as for troponin I and troponin T.

**Figure 5:**
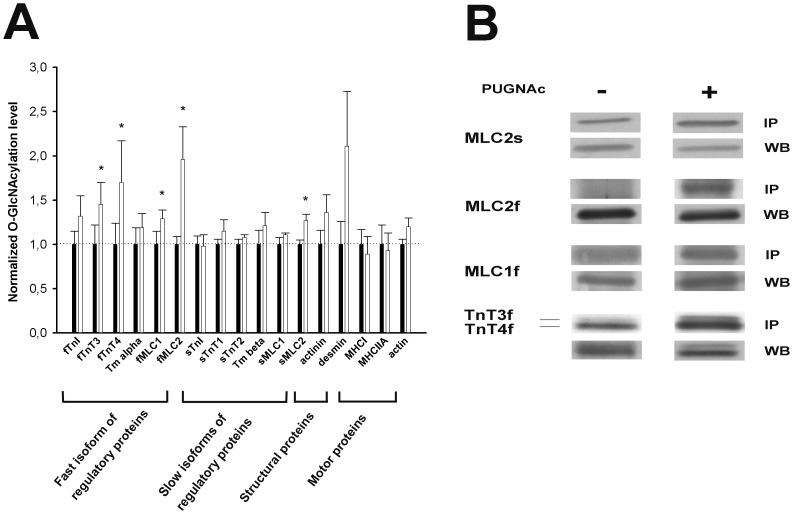
Quantification of O-GlcNAc variation on contractile protein after PUGNAc-treatment of soleus biopsies. Proteins extracted from skinned biopsies were RL-2 immunoprecipitated and separated on 10–20% SDS-PAGE. Densitometric analysis on QuantityOne software was performed on proteins of interest revealed using specific antibodies. (A) Results are expressed as ratio between signal obtained after RL-2 immunoprecipitation and signal obtained for whole protein level. Histograms correspond to O-GlcNAc level in PUGNAc-treated soleus comparing with untreated biopsies which were assigned to reference (*i.e.* 1). Data are expressed as mean ± SEM and are representative of 5 paired biopsies. *p<0.05 PUGNAc *vs* untreated-treated biopsies. (B) Representative western blots of the proteins presenting variation in O-GlcNAcylation after PUGNAc treatment showing the O-GlcNAcylated form (IP) and the total expression (WB) of the protein in control (−) and PUGNAc (+) treated biopsies. Only the region of the protein of interest is shown.

We measured the O-GlcNAc level in PUGNAc-treated and untreated soleus biopsies, untreated biopsies serving as reference; results are presented on Fig.5A. We have classified the studied proteins in four groups as being: the regulatory proteins (*i.e.* MLC2, MLC1, tropomyosin, TnT and TnI), with a distinction between fast and slow isoforms; the structural proteins (*i.e.* actinin and desmin); and the motor proteins (*i.e.* actin, MHCI and MHCIIA).

There is a slight but not significant increase of O-GlcNAcylation for alpha-tropomyosin, fast TnI, and for the structural proteins desmin and actinin alpha 2 (0.05<p<0.1). In contrast, we have measured a significant increase of O-GlcNAc level on five proteins (Fig. 5A) illustrated in Fig.5B: slow MLC2 (sMLC2) (+27.2% increase of O-GlcNAc level from the untreated state), fast MLC2 (fMLC2) (+96.8%), fMLC1 (+29.7%), fTnT3 (+45.43%) and fTnT4 (+70.8%). We excluded that the increase on O-GlcNAc level on these proteins resulted from an increase of O-GlcNAcylation on partner proteins since actin, myosin and tropomyosin did not present increase in their O-GlcNAcylation level.

**Figure 6:**
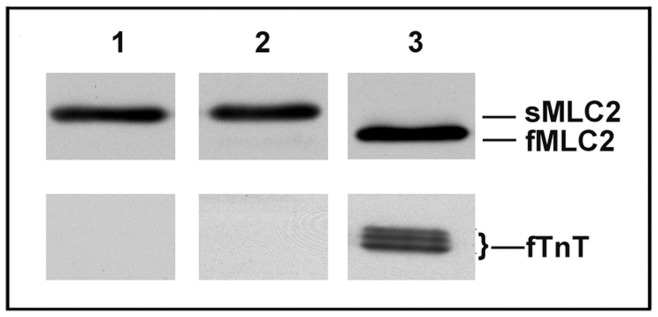
Western blot analysis of regulatory protein expression in slow and fast fibers (B) corresponds to western blot characterization of slow and fast isoforms of MLC2 (above) or TnT (below) for slow fiber isolated from untreated soleus (1), for slow fiber isolated from PUGNAc-treated soleus (2) or fast fiber isolated from epitroclearis muscle (3).

Interestingly, the increase of O-GlcNAc level affects preferentially all the fast isoforms of regulatory proteins, even if the fast isoforms are weakly expressed in soleus. Concerning the slow isoforms, predominantly expressed in the slow soleus muscle and exclusively expressed in slow fibers, we noted that the only significant increase of O-GlcNAc level occurs on myosin regulatory light chain. Preliminary to the application of increasing concentrations of calcium solution (T/pCa relationship establishment protocol), the fibers were tested with the strontium (as described in Material and Methods) to determine whether fiber was slow or fast, since 16% of soleus constitutive fibers being fast [29]. Only slow fibers were included in T/pCa analysis. The Fig.6 showed that the fibers included in the T/pCa relationship analysis expressed only slow TnT and MLC2 (lanes 1 and 2 on Fig.6) and not the fast TnT and MLC2 as it is shown for a fiber isolated from the fast epitrochlearis muscle (Fig.6, lane 3). This is the reason why the results obtained concerning T/pCa parameters were associated to the O-GlcNAcylation variation on slow isoforms only, since there are the only isoforms expressed in the slow fibers included in our calcium activation parameters analysis.

## Discussion

Our data clearly demonstrate that the increase in O-GlcNAcylation of soleus muscle fiber proteins, using two different inhibitors of O-GlcNAcase, is closely linked to an increase of calcium affinity. Proteomic identification of O-GlcNAc bearing proteins in skinned biopsies enabled us to determine that some of the proteins of the troponin complex, in particular TnI and TnT as well as some key structural proteins, actinin and desmin, were O-GlcNAcylated in addition to those previously identified [18], [19], [20]. The analysis of the O-GlcNAc level on these proteins permitted to determine those exhibiting an increase in their O-GlcNAcylation after treatment with PUGNAc. While we observed that fast isoforms of contractile proteins were more sensitive to an increase of O-GlcNAc level, we measured a significant increase of O-GlcNAcylation level on the slow MLC2, a protein playing an important role in the modulation of calcium activation parameters. Since the fibers used for functional experiments correspond to slow fibers, expressing only slow contractile proteins, the increase in MLC2 O-GlcNAcylation could be a candidate to explain the calcium activation parameters changes observed in hyperglycosylated fibers, without excluding that an unidentified O-GlcNAc proteins, such as TnC, could be potentially involved in this mechanism.

### 

#### Effect of the increase of O-GlcNAcylation on the calcium activation properties of soleus

The experiments performed on skinned fibers isolated from PUGNAc-treated soleus as well as from Thiamet-G-traited soleus clearly showed an increase in the calcium affinity, characterized by an increase of pCa_50_, while neither sensitivity (pCa threshold) nor cooperativity within the thin filament (n_H_) were modified. The analysis of CTD110.6 immunoreactivity pattern against the proteins of these skinned fibers clearly demonstrated that the modification in the calcium activation parameters was associated with an increase of O-GlcNAcylation on predominant proteins expressed in soleus skinned fibers. Recent data demonstrated that PUGNAc could have an effect by inhibiting lysosomal hexosaminidase [36], [37]. Since no complex glycans were detected on proteins extracted from skinned biopsies, we concluded that the only effect of PUGNAc on skinned fibers (and included in T/pCa experiments) was the increase of O-GlcNAc level. This was confirmed by the similar results obtained for Thiamet-G treated fibers.

To better understand the process involved in the modulation of the calcium affinity by O-GlcNAc, we identified from skinned biopsies the proteins presenting an increase in their O-GlcNAc level using a proteomic approach. We confirmed the O-GlcNAc modification of some contractile proteins such as the motor proteins, actin and MHC, tropomyosin, MLC1 and MLC2, as previously described [18], [19], [20] as well as proteins involved in the sarcomeric organisation such as the small heat-shock protein alphaB-crystallin. Two other key proteins of the sarcomeric structure, actinin and desmin, were newly identified to be O-GlcNAc. This approach was not sufficient to detect whether the constitutive proteins of the troponin complex were O-GlcNAcylated. Using immunoprecipitation experiments followed by western blot analysis, we determined the effective O-GlcNAcylation of some of component of the troponin complex, in particular TnI (slow and fast isoforms) and TnT (sTnT1, sTnT2, and fTnT1 to fTnT4). These data give new insights since among troponin complex proteins, only cardiac TnI was previously described to be O-GlcNAcylated [20].

### PUGNAc Leads Specifically to Increase of O-GlcNAc Level on Five Proteins, Especially on the Slow Isoform of Regulatory MLC

To assess whether the increase in calcium affinity might be associated to increase of contractile protein O-GlcNAcylation, we measured the O-GlcNAcylation variation on proteins identified in this study. In particular, our results strongly showed an increase of O-GlcNAc level on slow and fast MLC2, fMLC1, fTnT3 and fTnT4. We observed that the fast isoforms of regulatory proteins are preferentially affected by the PUGNAc-treatment. In the same way, higher global increase of O-GlcNAcylation pattern has been reported in fast epitrochlearis than in slow soleus muscle after treatment with PUGNAc [25], [38]. These data could result from differential regulation of O-GlcNAcylation between fast and slow muscles, at the whole cellular level as well as on isolated proteins.

However, our immunoblot analysis demonstrate that the slow skinned fibers used in functional experiments expressed only slow isoforms of regulatory proteins as previously reported [34], [35]. Thus, only the increase in slow MLC2 O-GlcNAcylation might be related to the modifications in calcium activation properties observed in hyperglycosylated fibers.

As previously reported (Hedou et al., 2007), sMLC2 was detected in the flow-through fractions of WGA-immobilized affinity chromatography demonstrating that only one part of this protein was modified. We measured on these experiments that 24,27±3,37% (n = 3) of total sMLC2 was O-GlcNAcylated. Even if the 25% increase of sMLC2 glycosylation would represent a slight increase (6.25%) we can consider that it could be not negligible in the modulation of contractile activity. Thus increase in O-GlcNAcylation of actin from 27.6% to 35.1% (n = 4) was associated to changes in calcium affinity of skinned fibers (Ramirez-Corea et al., 2008). Previous studies revealed that phosphorylation of MLC2, catalysed by a Ca^2+^/calmodulin-dependant MLC kinase, increases the force development at submaximal calcium concentration, conferring a higher calcium sensitivity to the fibers [39], [40], [41], [42]. Thus, O-GlcNAcylation or phosphorylation of MLC2 could have functional consequences on calcium sensitivity, as it was previously proposed in cardiac muscles [20].

However, we cannot totally excluded that another O-GlcNAcylated proteins could be involved in the modification of calcium affinity. One candidate could be TnC which plays a key role in the modulation of the Ca^2+^-activation characteristics of skinned muscle fibers. However we could not identify TnC as an O-GlcNAcylated protein, and in our knowledge, TnC was never identified to be O-GlcNAc modified in skeletal muscle nor in cardiac muscle.

### Conclusions

In summary, recent works realized in striated muscle strongly associate O-GlcNAcylation with the modulation of calcium activation parameters. In this paper, we reinforce the idea that O-GlcNAc might be involved in the regulation of skeletal muscle contraction, since the increase of O-GlcNAcylation on contractile protein patterns leads to an increase of calcium sensitivity. The regulatory MLC2 could be a good candidate to explain this effect. This particular interplay between phosphorylation and O-GlcNAcylation remains poorly understood, and it will be interesting in the future to consider the interplay between phosphorylation and O-GlcNAcylation on MLC2 in muscle disorders (aging, skeletal muscle functional atrophy, etc…) presenting variations in calcium activation parameters to provide new data to understand the physiopathology of such diseases.

## Supporting Information

Figure S1 **Effect of the protocol of incubation on calcium activation parameters of skinned fibers isolated from soleus**. (A) T/pCa curves were representative of 6 fibers from control skinned biopsies (•) and 6 fibers from inbubated skinned biopsies (○). Data were presented as mean ± SEM. Curves were fitted with the Hill parameter.
(TIF)
