# Supplementary figures and images for: Increasing O-GlcNAcylation Level on Organ Culture of Soleus Modulates the Calcium Activation Parameters of Muscle Fibers
Source: PLoS One. 2012 Oct 24;7(10):e48218. doi: 10.1371/journal.pone.0048218 (PMC3480486; doi:10.1371/journal.pone.0048218)

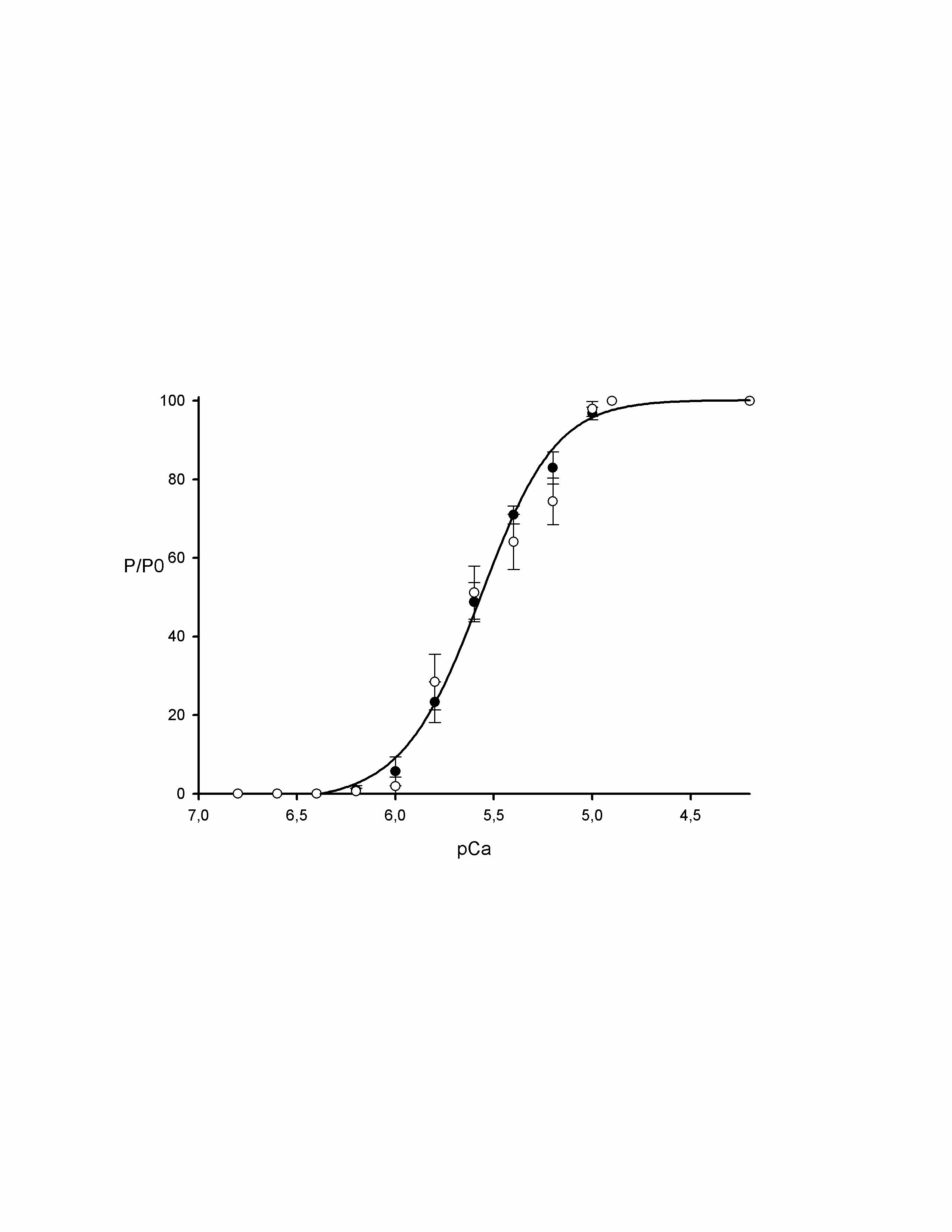

Supplement: Figure S1 — Effect of the protocol of incubation on calcium activation parameters of skinned fibers isolated from soleus. (A) T/pCa curves were representative of 6 fibers from control skinned biopsies (•) and 6 fibers from inbubated skinned biopsies (○). Data were presented as mean ± SEM. Curves were fitted with the Hill parameter. (TIF) [file pone.0048218.s001.tif]
